# Youth collective action for accountability towards sexual and reproductive health (SRH) rights: a systematic scoping review

**DOI:** 10.1186/s12889-026-26642-8

**Published:** 2026-02-26

**Authors:** Sana Q. Contractor, Ana Lorena Ruano, Devaki Nambiar, Sara Van Belle

**Affiliations:** 1 Centre for International Health, University of Bergen, Overlege Danielssens Hus, Årstadveien 21, Bergen, 5009 Norway; 2 Institute of Tropical Medicine, Nationalestraat 155, Antwerp, 2000 Belgium; 3 The George Institute for Global Health, 308, Third Floor, Elegance Tower, District Centre, Jasola Vihar, New Delhi, Delhi 110025 India

**Keywords:** Youth, Youth health, Social accountability, Social movements, Participation, Participatory action research, SRHR

## Abstract

**Background:**

In the field of sexual and reproductive health and rights (SRHR), there is a growing consensus that participation is critical to ensuring that the voices of youth are heard and their agency is exercised in a way that enables them to practice active citizenship. This includes collective action to hold duty bearers and power holders to account for their rights. Youth are increasingly being seen as important constituents; however, despite the growth in practice, the evidence on what strategies are adopted, how these initiatives work and why is limited.

**Objectives:**

To explore interventions implemented within the ambit of accountability for SRHR rights, and to understand how these interventions generate or strengthen collective action to ultimately lead to the improvement of sexual and reproductive health and rights.

**Methods:**

We conducted a scoping review following the 5-step methodology developed by Arksey and O’Malley. Systematic searches were conducted on PubMed and Web of Science databases, identifying articles from Jan 2008 to Dec 2022, about interventions with youth that involve collective action for accountability pertaining to SRH rights. Data were charted, synthesized and reported.

**Results:**

A total of 25 articles describing 18 interventions were included and represent a range of interventions categorised as collective action. The interventions can be divided into three categories: first, articles analysing the impact of community-based interventions by NGOs or external agencies (15); second, articles describing the evolution and impact of advocacy networks and broader social movements (4); and articles examining (national or global) policy advocacy processes that seek to engage youth (6). The strategies are analysed across three broad categories/themes, identifying what factors influenced the success of each: (1) Building youth empowerment and solidarity – seeding youth collectives as critical mass, ensuring diversity in the collectives, and the extent of critical consciousness and analysis engaged in by them (2). Building pressure and allyship – casting a wide net of allies across different interest groups, allying with adults in a manner that fosters equitable collaboration, building strategically relevant evidence and framing issues in a manner that speaks to change-makers and people at large; and (3) Negotiation with power holders – going beyond the state and negotiating with other power holders, building on existing favourable legislation, utilizing invited spaces and leveraging of political opportunities.

**Conclusions:**

As the interest in youth leadership in SRH deepens, this review underscores that even in contexts where youth engagement is recognised, the readiness of the system to respond to and grant legitimacy to youth voices is necessary for effective implementation. The evidence suggests that collective action interventions must be multi-level, strategic, and consider local social hierarchies, histories of civic organising, and policymaking. Power holders, not just at the state but also at the community level, must be held accountable for the sexual and reproductive rights of youth and their leadership to be realised.

**Supplementary Information:**

The online version contains supplementary material available at 10.1186/s12889-026-26642-8.

## Introduction

Youth participation was first recognised as germane to SRH at the International Conference on Population and Development (ICPD) in 1994 [1, 2]. Since then, there has been a growing consensus that participation is critical to ensuring that the voices of youth are heard and their agency is exercised in a way that enables them to practice active citizenship. It is of vital importance because of its status as a right in itself, and has also been considered one of the factors that may influence the success of SRH programs and policies [3]. It includes the right to participate in decisions pertaining to the personal, such as bodily autonomy, sexuality and the choice to marry; as well as the public, such as influencing policies and programs affecting these. Efforts to ensure the participation of youth in policies and programs can take various forms, including through education and capacity building, creating platforms for dialogue, consultations to inform decision making vis-à-vis health services, programs and policies, including youth in the design, implementation and monitoring of programs and interventions. Youth may also organise themselves, or be organised, to take social action within their communities [3]. When actions to demand accountability are carried out by citizens or communities, this is referred to as “social accountability” [4], and may take the form of collective action - that is, an effort to bring about social or political change through a coordinated group [5]. This includes holding duty bearers and power holders accountable for their rights as citizens.

Communities, especially women’s groups, coming together to engage in collective action in order to demand accountability for health entitlements, have proven to be an important intervention to improve SRH rights [6]. Reported impacts range from improvement in health outcomes, health system responsiveness, provider responsiveness, and SRH priority setting, among others [6]. The focus on youth engagement in these strategies has gained traction in recent years as they are recognized as an important group with unique needs and potential in addressing SRH challenges [3]. Youth are also increasingly being seen as an important constituency in global and national processes of policy advocacy and at platforms where accountability can be sought. For instance, the Global Forum for Adolescents, hosted by the World Health Organisation - Partnership for Maternal, Newborn and Child Health in October 2023, was a platform designed and hosted by youth to take stock of progress and promises made towards achieving the SDGs from their perspective. However, despite the growth in practice in these areas, the evidence on types of collective action interventions implemented with youth for SRH accountability is limited. *What strategies are adopted by such interventions*,* who is involved*,* how they work and why*,* is not sufficiently known.* This review aims to explore the kinds of interventions that have been implemented within the ambit of collective action and accountability for SRH rights, and to understand how these interventions generate or strengthen collective action to ultimately lead to the realisation of sexual and reproductive health and rights.

## Methods

We conducted a scoping review to identify the evidence on collective action and accountability interventions in the domain of SRH rights, the approaches or study designs that have been used, and how the studies explain the changes taking place due to the intervention in their specific contexts. This helps to identify gaps in the literature and suggest future areas of study to better understand how and when collective action interventions with youth work or do not work. Scoping reviews are recommended to examine how research has been conducted on a certain theme and to identify research gaps [7]. 

We conducted the scoping review following the 5-step methodology developed by Arksey and O’Malley [8]. This includes (i) Identifying the research question (ii) Identifying studies relevant to the review (iii) selecting studies to be included in the review (iv) charting the data and (v) collating and reporting results. These steps have been elaborated upon below. The PRISMA checklist for scoping reviews is attached as supplementary file 1. A review protocol was prepared but is not registered.

### Identifying the research questions

This scoping review answers the following questions:

1. What are the types of collective action/accountability interventions have been implemented with adolescents and/or youth to improve Sexual and Reproductive Health Rights?
2. What are the theoretical/conceptual frameworks underlying these interventions?
3. What strategies do these interventions adopt?
4. What facilitates or hinders change?

### Identifying studies relevant to the review

Using the research questions of the review as a starting point, we conducted a search using “youth,” “collective action,” “accountability”, and “sexual and reproductive health rights” (and related terms) as search terms. However, after an initial reading of the literature, we decided to broaden the scope of the search strategy by looking for related concepts in the literature, such as youth participation, social movements, advocacy, citizenship, and social accountability. (Table 1)

**Table 1:** Search terms (columns connected by AND, rows connected by OR)

SRH	Age	Intervention
Sexual and Reproductive Health	Young people	Youth participation
Reproductive health	Youth	Social Accountability
Family Planning	Adolescents	Accountability
Contraception	Girls	Collective Action
Abortion		Citizenship
Sexuality Education		Civil Society
Maternal Health		Advocacy
Adolescent SRH		Social Movements

A full electronic search strategy is attached as Supplementary file 2.

We searched the databases of PubMed and Web of Science (WoS) to cover not only public health but also applied social sciences, psychology, youth studies, and development studies. This draws on the recommendation of previous reviews, which find that literature from other fields (such as youth studies) has relevance to accountability as a concept and can add value to our analysis [6]. Finally, a search on Google Scholar was done to identify articles from the grey literature using the terms “youth”, “collective action” “participation” “accountability” and “SRH”.

### Selecting studies to be included in the review

The search strategy identified 1527 citations (775 WoS + 862 PubMed, with 112 duplicates). A total of 1420 articles were eliminated as they were out of the scope of the review question. We screened the remaining 107 full texts to include only those articles that referred to collective action interventions like programs that organise youth/adolescents to advocate for their rights. This includes addressing a power holder (within or outside the community) whom the group confronts/challenges and from whom it demands accountability. We included a range of interventions that fit the criteria of “collective action” – including interventions implemented by development organisations, social movements, and youth efforts in policy advocacy – because these would expand the current understanding of interventions and how they work in different situations. This round of screening was done by two authors. Inclusion and exclusion criteria are elaborated upon in Table 2.

**Table 2:** Inclusion/exclusion criteria

Criteria	Include	Exclude
Language	English	Other languages
Countries	All	All
Period	1 st January 2008-31st December 2022	Before 2008
Publication type	Peer-reviewed research, grey literature, NGO reports	Commentaries, editorials, letters, reviews
Study design	All	---
Study group	Adolescents and youth	Non-youth Interventions that work only with young men/boys^a^
Intervention of interest	Adolescents or Young People are part of a collective action intervention, demanding accountability Intervention addresses sexual and reproductive health rights	Youth participation interventions that do not involve collective action and accountability from power holders Interventions that only educate or raise awareness among youth. Provider/stakeholder perspectives on youth participation
Quality of information	Analytical articles, with explanations/reflections on what worked and why	Articles describing the intervention and outcomes, but no reflection or explanation

^a^We did not include interventions that only work with young men and boys, because the theory of change for that demographic would be very different. In the context of Sexual and Reproductive Rights, rather than “empowerment”, interventions with boys are typically meant to facilitate recognizing and letting go of privilege. This process of change is very different to the one with girls and young women and is beyond the scope of this paper

**Fig. 1:**
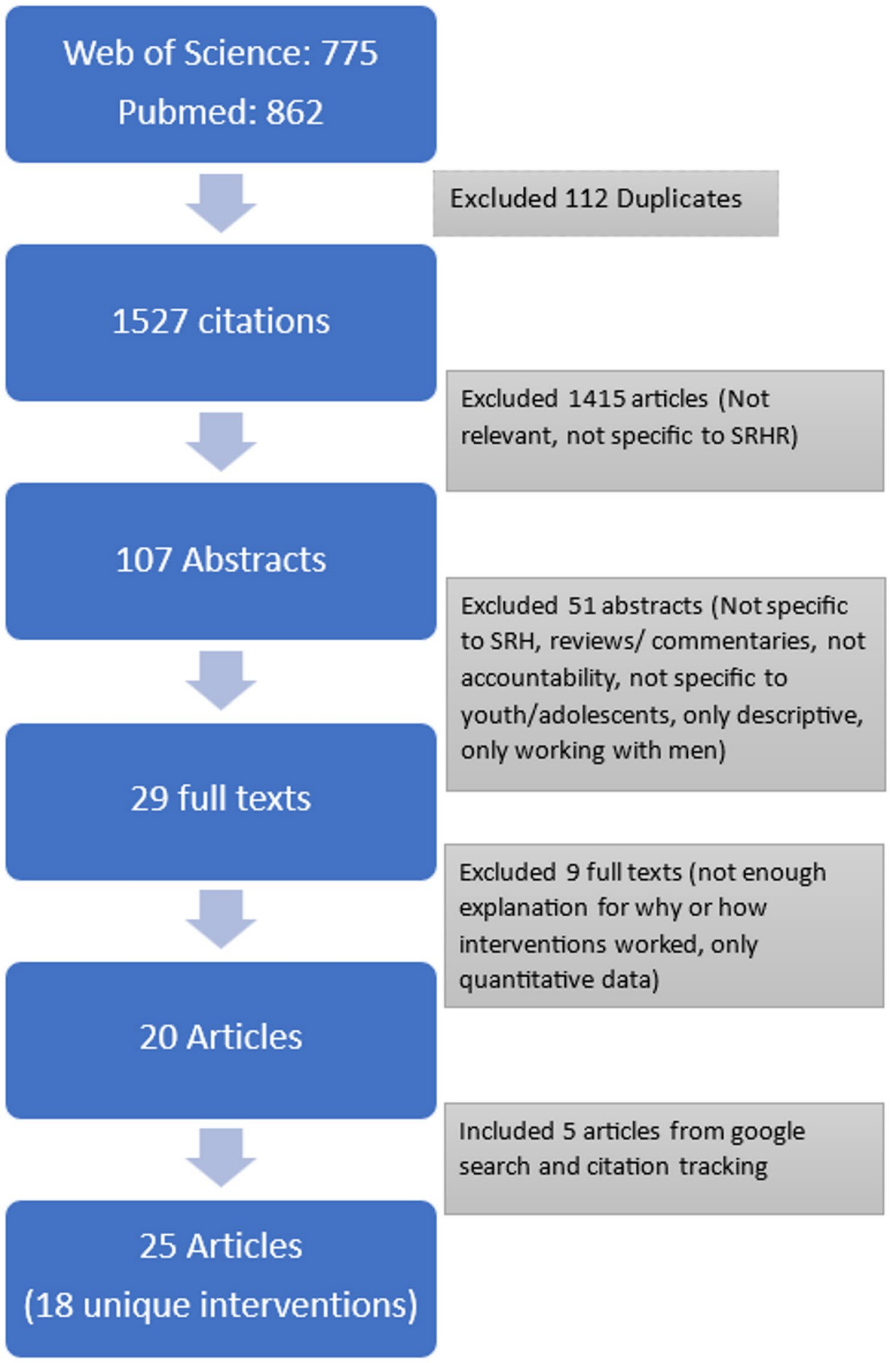
Selecting articles

Finally, we included articles from grey literature in the review by (1) conducting a search on Google Scholar (first ten pages) and (2) scanning references from review articles/reports. (See Fig. 1 for a flowchart depicting how articles were screened)

### Data charting

The selected studies were then analysed and charted in a data extraction sheet, which was vetted by SQC and SVB. This included (1) study characteristics such as title, year of publication, type of publication, research methodology, country, and type of SRH issue addressed and (2) description of the intervention, its conceptual underpinnings, and (3) reflections and learnings relevant to the review. (An edited version of this sheet is included as Supplementary file 3)

### Collating and reporting

The collating of data focused on identifying the findings with explanatory potential within three broad strategies (1) Building youth empowerment and solidarity (2) Building pressure through evidence and allies (3) Negotiation with power holders, informed by prior work of authors SVB and SQC [6]. The findings are described to answer the original research questions of the review. Finally, in the discussion, gaps in the research were identified and contextualised against broader concerns in the field of youth collective action.

## Results/findings

This section is a synthesis of the findings emerging from the articles included in the review. We begin by describing the particulars of the articles that were included and the types of interventions that they include. We then synthesise the various rationales that the articles provide for engaging youth, and the conceptual frameworks that guide the interventions. Finally, we describe the strategies employed by the interventions – the Theory of Change – along with an analysis of what factors enable or hinder the success of the interventions. Details of the interventions are provided in Supplementary file 3.

### Study characteristics

A total of 25 articles describing 18 interventions^1^ were finally included in the review. Of these, 22 articles were from peer-reviewed journals, and 3 were reports of a multi-country NGO (Non-Governmental Organisation) program. All the articles used qualitative methods, relying on data collection methods such as focus groups and in-depth interviews. A few (N=?) also relied on project documents, direct observations of interventions, and the diaries or fieldnotes of project workers. Only two [9, 10] used mixed-methods, primarily to assess the impact of the intervention in terms of service uptake or changed behaviour.

In terms of geographical scope, the interventions were global as well as national and local. The interventions were distributed across the continents of Asia [2], Africa [11], and the Americas [3]. Two articles examined processes at the global level. In terms of income groups, 10 interventions were from low and low-middle income countries and 6 were from upper-middle- or high-income countries.

### Types of interventions

The articles represent a range of interventions that can be categorised as “collective action.” They are broadly divided into three categories: first, articles analysing the impact of community-based interventions implemented by NGOs or external agencies [15], second, articles describing the evolution and impact of social movements and advocacy networks [4], and articles examining global, national, and local policy advocacy processes that seek to engage youth [6]. The first category includes interventions consisting of participatory action research projects in which researchers are involved in seeding the intervention and learning from it, bounded NGO projects that deploy accountability tools like score cards or photovoice, or longer-term NGO-run initiatives that include a range of interconnected strategies and address a number of issues over a long period of time, usually more than a decade. The second category includes networks of social movements [11–13]. The third examine national and global efforts to increase youth participation in policy development, and youth participation in invited government spaces like committees [14–19]. Of these, only two articles in the first category [20, 21] included only girls/young women, whereas all the others addressed [22] both boys/young men and girls/young women. (An overview of the articles and interventions is provided in Table 3.)

**Table 3:** Types of interventions and SRH issues addressed

	Type of intervention	Brief description	SRH issues	Citation
1	Community-based interventions by NGOs/external agencies	Youth Action Research for Prevention (YARP) in urban United States	Urban youth issues, specifically addressing “risky sex” and drug use among youth.	[10]
2		Youth engagement in HIV management in South Africa	HIV/AIDS	[23, 24]
3		Impact of Community Score Card on addressing issues of pregnant adolescents in Uganda	Maternal Health	[20]
4		Long-term NGO intervention with multiple iterative cycles in India	SRH + intersectional issues of disabled and transpersons	[25]
5		Long-term International NGO intervention using Community Score Cards in Malawi	Adolescent SRH	[26, 27]
6		Youth driven HIV prevention initiative using Photovoice in Malawi	HIV/AIDS	[28, 29]
7		Youth- led participatory Action Research to address rape culture with privileged girls in New York, United States	Violence Against Women	[21]
8		Youth-led PAR to address ASRH issues in Senegal	Adolescent SRH	[30]
9		Community Score Card intervention through Youth Working Groups to address Contraception Access in Kenya	Contraception	[9]
10		Youth-led social accountability processes using CSC and Client feedback in Malawi, Ethiopia, and Ghana	Youth SRH	[31–33]
11	Social movements and advocacy networks	Network of organizations advocating for sexual health policy in Ecuador and Peru	Sexual Health	[12, 13]
12		Youth Collective Action for an abortion law in Rwanda	Safe Abortion	[11]
13		Youth Activism for LGBTQ rights in Indonesia	Queer rights	[22]
14	Youth participation in global/national/local governance	Engaging young people in decision-making at UNAIDS	HIV/AIDS	[15]
15		Youth leadership in transnational advocacy spaces	Population and climate change	[17]
16		Experiences of youth with SRH policy making spaces in Malawi	Youth SRH	[18, 19]
17		Youth participation in health governance bodies at the community level in Kenya	Youth SRH	[14]
18		Youth Participation in the Adolescent and Youth Health Policy in South Africa	Youth and Adolescent Health	[16]

### Rationale/motivations for working with youth

The articles included in this review describe a range of motivations for working with youth that are not mutually exclusive and usually used in tandem. The most common rationale is the principle of the human right to participation. Many articles make mention of the fact that human rights instruments, ICPD, and some regional and national treaties prescribe the obligation to ensure youth participation in decisions affecting them [11, 15, 16, 22, 23, 25]. Beyond the formal human rights framework, almost all the articles recognise both gender equality and SRH as rights of youth, this being one of the major reasons for engaging youth in SRH programs and advocacy. The second most stated rationale for working with youth emphasises the importance of their participation in better and more effective implementation of programs or policies. This could include, for example, concerns around equity and the fact that adolescents or young people are left out of reproductive health programs [9, 20], the need to factor in youth perspectives in policy formulation in South Africa to give it legitimacy [16], ensure sustainability of previously existing initiatives, such as a community score card intervention in Malawi [27], or more instrumentally to address youth-related factors in programs to prevent HIV [14, 28]. 

Other rationales include what Berg et al. refer to as the advantages of the developmental stage. It is in this period that adolescents seek more autonomy and build their opinions, which makes it an opportune time to intervene [10]. Mecwan et al. refer to their value as “demographic dividend” which can be seen as a resource to be leveraged for the future [25], and Sasser alludes to how youth leadership is encouraged because they are seen as having the potential to “turn the tide” with respect to population growth and climate change [17]. Mwale, Mecwan and Campbell mention the potential that youth engagement in SRH can have on deepening democratic engagement and citizenship efforts of youth more broadly [23, 25, 26]. Coe et al. [13] also see the emergence of youth leadership in a social movement for sexual health in Latin Peru and Ecuador as emerging from adult-seeded work, but re-invented as a “a new generation of activism on body politics among young people”.

### Theoretical lens of the interventions

Drawing on the rationale of youth participation as an imperative, most of the articles refer to human rights frameworks or global development initiatives as guiding the collective action intervention to secure entitlements. Some go beyond this to explicitly mention a conceptual framework or a theory/philosophy underpinning the intervention. Four articles mention interventions using Youth Action Research or Youth Participatory Action Research, which consists of a cycle of capacity building, youth-led research on a specific issue identified by the youth, and the use of this research for social action [10, 21, 23, 30]. However, two further qualify it with a conceptual framework or philosophy that informed Youth Participatory Action Research (YPAR) [10, 21]. Proweller et al. [21] used a pedagogy that drew on critical intersectional feminist principles and community-engaged action toward social justice, which directs attention to the root causes of social problems. This created an environment where young women could “intentionally draw on a feminist approach to interrogation and critique through which to locate themselves within intersecting axes of privilege and oppression.” Berg et al. [10], working with disempowered urban youth in the United States, used a Youth Participatory Action Research (YPAR) methodology, informed by a socio-ecological theory which situates individuals within social systems rather than intervening at the level of individual alone; identity theory which allows youth to reflect on the construction of their identities and its adaptation in different settings; social learning theory is the basis of group formation and provides the platform for developing interpersonal skills and mutual exchange of ideas; and critical theories which focus on structural barriers to gender equity and transformation at the individual group community and structural levels. Within the latter, they drew upon Foucault’s work on power, Bourdieu’s work on structures of oppression, and Freire’s work on the ability of those living in oppressed conditions to drive change by conducting a transformative analysis of their circumstances. They connected this analysis with action and empowerment through Gramsci’s work on maintaining hope in the face of structural barriers, as they saw this as particularly useful when working with youth who might initially sense great powerlessness when they analyse their surroundings. Lofton et al. use photovoice methodology which is a method that involves using photographs of community realities, as a starting point for critical reflection, dialogue and action. Although photovoice is a tool, it falls within the ambit of PAR because it utilises experiential knowledge of communities to trigger dialogue/discussion and action [28]. Mecwan et al. draw on different philosophies, including the recognition of gender inequalities in society, intersectionality, or the recognition that multiple axes of disadvantage exist, a rights-based approach that guarantees sexual and reproductive health as a responsibility of duty bearers, and social accountability or the collective demand for entitlements guaranteed as human rights [25]. 

Articles evaluating the engagement of youth in policy-making and advocacy spaces are informed by theoretical frameworks that allow for critical analysis. Evelia et al. refer to Harts’s “ladder of youth participation” (drawing on Arnstein’s ladder of citizen participation), Treseder’s “degrees of participation” and the CHOICE “Flower of participation” to analyse meaningful youth participation in governance processes [14]. Sasser uses a lens of “technologizing of women’s rights” to critically analyse youth participation in global advocacy spaces [17]. Jacobs and George et al. use the domains of Place, Purpose, People, Process and Partnerships, adapted and expanded from Cahill and Dadvand’s youth participation framework, to guide an analysis of the same in policy-making in South Africa [16]. Wigle et al. use postcolonial feminist and difference-centred citizenship theory to analyse the experiences of diverse youth in participating in governance processes [18]. Coe et al. build and expand upon Tilly’s concept of “tactical repertoire” to explain the dynamics of youth activism in Peru and Ecuador [13]. 

### Theory of change for collective action – why and how do strategies work/do not work

The broad contours of the theory of change for the interventions can be understood from the strategies employed by them. Broadly, collective action efforts that seek to hold power holders accountable, involve some kind of mobilisation and capacity building of youth, leading to them becoming informed agents of change. They critically analyse their situation and surroundings to identify key problems/challenges, which they then address as a group and/or with allies, which gives them the collective power to raise issues and negotiate with power holders to bring about change. The articles included in this review largely adhere to this cycle but may also focus more specifically on certain parts of it. For instance, those who examine youth participation initiatives in policy development are more concerned with the aspect of negotiation, but they also allude to the other parts of this process of change-making.

**Fig. 2:**
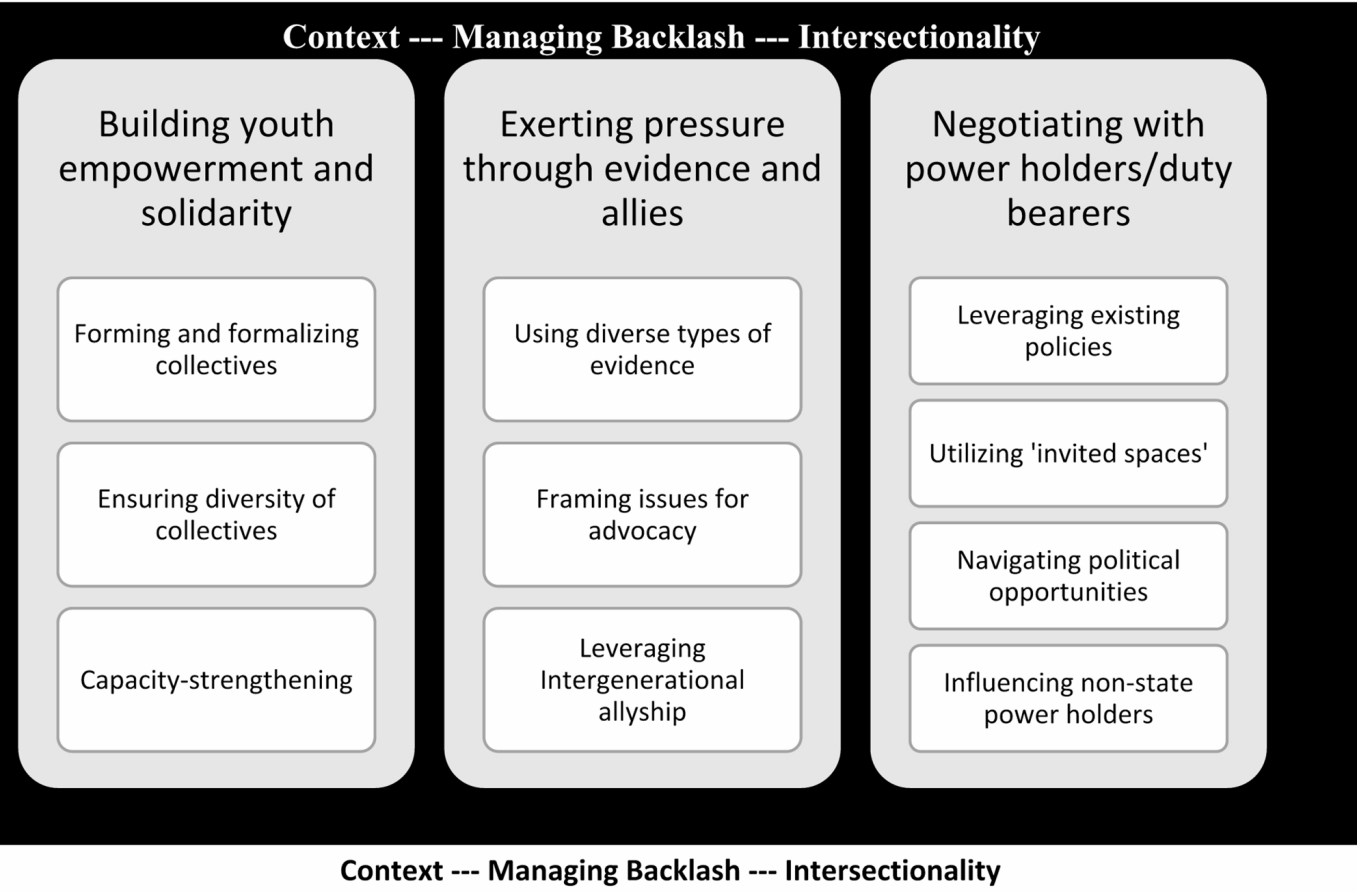
Strategies for youth collective action on SRH

In the subsequent sections, we discuss these strategies, dividing them into three broad categories: (1) Building youth empowerment and solidarity (2), Building pressure through evidence and allies, and (3) Negotiation with power holders. In each section, we will describe the diversity of strategies employed in different contexts, and the effectiveness or drawbacks of each as described in the articles. (For a graphical depiction of strategies and emerging themes in each, see Fig. 2)

#### Building youth empowerment and solidarity

The building of an empowered collective that can identify and articulate issues of youth, is a prerequisite for effective collective action. The community-based interventions and advocacy network/social movement interventions both describe a process of collective building, while the articles on youth involvement in policy advocacy and governance emphasise the need for youth collectives.

##### Formation of youth collectives

A commonly used strategy is the creation of platforms for youth engagement, such as groups or meeting spaces. The context affects how and with whom these groups are made. While some interventions prefer single-sex groups to make it easier to have conversations around sensitive subjects like SRH [29], others like Mecwan et al. insist on mixed groups, and that youth can speak and share their experiences at mixed-group meetings is seen as a positive outcome of the intervention [25]. Another intervention in Senegal [30] used the platform of the collective as a way to foster trust between ethnic communities by creating groups with mixed ethnicities. Some interventions that are looking to effect change in specific groups, build more homogenous groups – for instance, a youth participatory action research intervention in New York City that engaged privileged Jewish girls to question certain social norms around rape culture in their community. Similarly, Macwan et al. describe the creation of groups to specifically address unique issues of disabled and trans groups [25]. In case of LGBT groups, service provision is an entry point for organising because the issue is stigmatised and hence a need-based approach to reaching people is required. The communities are dealing with everyday violence and barriers in accessing identity cards, which makes it imperative for organisations to provide services rather than only engage in advocacy [22]. On the other hand, sometimes interventions focused on a specific health problem include youth in community groups as an important constituency, but their age and vulnerability (like being pregnant adolescents, for instance) have been reported as a barrier to participation [14, 20]. The outcomes of such collective building are both individual and collective. For instance, youth report being better informed, feeling “bolder,” and more confident [31, 32]. But going further, Coe et al. find that being involved as leaders in the network provided youth with an alternative to other potentially destructive engagements. They were able to engage with other youth from different backgrounds as well as other adults and this changed the way they thought about sexual health and human rights more broadly. At the collective level, having a group gave young activists legitimate positions and structures from which to carry out their advocacy [13]. 

##### Diversity of youth in collectives

The representation of youth in all their diversity is essential to build a movement for collective action. This was highlighted by Jacobs et al., who found that in South Africa, youth participation tended to be through “ambassadors” rather than an “active youth citizenry.” This is a legacy of the preceding youth engagement programs that created “celebrities” to raise awareness for HIV prevention. They state that this format of youth participation was without representation or accountability to the broader population of young people, and it did not attend to structural and systemic issues of youth disempowerment. They conclude that a “mobilised, capacitated, diverse youth citizenry” is essential for youth participation to truly be meaningful and effective. However, gender and other social and financial barriers limit the participation of diverse youth in collectives. For instance, Wigle [18] found that girls in Senegal who participated in the groups were seen as “prostitutes”. She also points out the impact that age, sex, marital status, and geographic location, on their participation*- “younger youth*,* females*,* people with limited formal education*,* and those living in rural areas have fewer opportunities and resources to engage meaningfully.”* This is echoed by Evelia et al. [14] who also find that because participation in the groups was voluntary, those without financial resources were less likely to attend. Moreover, adult members of health committees preferred not to include young women because it was perceived that they would marry and move away. In Ghana [32], those who could not read, for instance, were not made part of the groups. Biases were also seen based on clan fidelity and political affiliations.

An attention to diversity in building allies was highlighted in some articles. Coe et al. pointed out the importance of involving diverse youth groups from different social strata, finding common ground in their identity as “youth.” By doing this, the networks in Ecuador were able to bridge differences among different groups of youth: “mestizo” and indigenous, urban, and rural, as well as formally organised and organically emerging subcultures [13]. Similarly, Hildebrand (2013) found in an effort that was rolled out both on and offline to involve youth in decision-making processes on HIV/AIDS, responses for what change is required was different in online and offline groups in the same country – while offline forums emphasized the need for morals and more “responsible” behaviour, online forums focussed on opening up communication and breaking taboos surrounding sex and relationships [15]. This too highlights that youth opinions and demands are not homogeneous, nor are they always progressive. Hence, casting a wide net for allies and being judicious in making alliances is important.

##### Capacity strengthening of collectives

Bringing together a group of youth must be followed by a process of “empowerment”, which may include capacity building and/or a facilitated process that involves learning and reflection. In the articles reviewed, this process varies across interventions. Adolescence is seen to be a transition period when youth are experimenting with their behaviour and values while seeking to forge social connections with each other and the outside world. They are looking to exercise the agency that these capacity-building initiatives seek to build on [34]. While some interventions are more specifically focused on technical aspects of SRH [30, 31], some emphasise governance and leadership to equip youth in navigating the health system, especially if they are in invited spaces within the health system [25]. Sometimes, however, interventions may be narrowly focused, and their framing can amplify more biomedical interpretations of problems. For example, in Lofton’s study from Malawi, “risky” youth behaviours were defined as unprotected sex (without a condom), sex with multiple sex partners, or sex in a high-risk context, such as when alcohol is involved [28]. As a result, youth suggested solutions to reduce sexual encounters between boys and girls where they felt such encounters were likely. With regard to sexual and reproductive health specifically, Campbell et al. point out that the perspective with which dialogue is conducted in youth groups must be aligned with their reality [23], especially since this may be at odds with adult notions of sexual morality. Broader social contexts that promote abstinence may ultimately make it difficult to bring about change, but organisations must create safe spaces for conversations with youth. They therefore identify not just knowledge but critical thinking and ownership as important factors that influence the success of youth involvement initiatives. Others like Berg or Proweller use a broader social justice/feminist pedagogical approach that is informed by local historical, political and social realities, with the understanding that engaging in such critical analysis and reflection itself is a process of empowerment for youth [10, 21]. 

The articles highlight both the need to create a strong collective with a critical mass, but also the challenges and shortcomings that plague projects in being able to do so. While the creation of groups is a commonly used strategy, the extent of analysis that the group engages in varies greatly between interventions, with some being focused on the SRH policies that the program seeks to monitor/improve implementation of, to those that are aiming to build empowered citizens, to those that generate social movements. The inclusion of diverse persons in these collectives, although necessary, is also challenging and dependent on political and social contexts.

#### Building pressure through evidence and allies

To bring about the changes that it envisages, the collective must create pressure on power holders. This is typically done through generating evidence about the problem, onboarding allies with similar goals, and influencing public discourse and opinion. The latter is particularly important because, as Coe et al. argue, youth movements not only direct their efforts at the state but also play a critical role in bringing about discursive change and building organisations and networks as well [13]. This section refers to different ways in which the youth collective action interventions build/raise pressure to advocate for their demands and build broad alliances.

##### Diverse types of evidence

The articles describe a range of strategies that the interventions use to build evidence, taking into account what type of evidence youth could be engaged in gathering, what might be most effective, and what might represent different viewpoints. Evidence has been built through the use of score cards, client feedback forms, and photovoice by some interventions [9, 25, 26, 29, 32, 33]. While in some cases, youth were involved in designing the score cards, such as in CARE’s CSC intervention in Malawi [26], in other cases, the youth did not participate in this process [33]. Other articles mention the use of testimonies of those affected by rights violations. For instance, the policy advocacy for safe abortion in Rwanda gathered testimonies of young Rwandan women in prison for abortion and the extent of unsafe abortion in the country, which “put a face to the problem.” [11] Yet others have used online forums to crowdsource opinions and demands for change from the perspective of youth, but complemented it with offline consultations in order to provide more opportunities for participation and gathering of diverse viewpoints [15]. 

##### Framing issues for advocacy

Taking the evidence or the specific understanding of the problem of youth to a larger audience is a common strategy applied in many interventions. In some cases, this is done through organising debates or value clarification exercises to foster dialogue about the issues like abortion that are contentious, launching petitions, and using various news and cultural media to carry the message [11]. Building alliances or “social advocacy” also helps to broaden the range of stakeholders (e.g. communities, other youth groups) concerned with the problem, who might be able to lend their support to the issue. The strategic messaging required to bring about larger change is an important determinant of success. For instance, in the case of LGBT rights in Indonesia, activists felt that instead of only focusing on a rights-based approach, which is seen as “western,” the same agenda could be repackaged with messaging around “anti-violence”, which is a more acceptable discourse [22]. 

Sometimes, the combination of factors that it takes to propel advocacy efforts may act in perverse ways as well. For instance, Sasser (2014), describing the resurgence of the population control movement through “youth leadership”, points out that an oversimplified narrative propagated by young climate change activists with the discourse of “meeting the SRHR needs of young people around the world can help stabilise population and contribute to comprehensive strategies to reduce CO2 emission.” This, coupled with imperfect data, has allowed organisations to instrumentalise youth engagement. This resonates with Sukareih and Tannocks [35] appeal to critically view both the negative and positive portrayals of youth engagement. To analyse unintended consequences such as the instrumentalisation of youth engagement.

##### Allyship with adults

Deliberate efforts to build allyship with adults were mentioned in several articles. Many studies highlight the negative views of adults about youth as a barrier to successful program implementation. While Campbell et al. (2019 found reluctance by community adults to recognise the potential value of youth inputs, promoting abstinence, and an unwillingness to regard youth as equals in project structures to be significant barriers, many studies also see a more constructive role for adults. Jacobs and George describe the important role played by trust relationships between youth, academics, and policy makers in furthering meaningful youth engagement, which were built through long-standing participatory action research projects in South Africa [16]. Sometimes adults are also seen as mentors [13, 21, 28, 30]. Adult organisations may mentor and coach younger leaders who are inspired and whose capacity can be strengthened to form their own organizations/articulations/collectives. In the case of Coe et al., seeing an adult leader/being exposed to a particular activity gave young people inspiration to pursue certain professions/vocations. (e.g. participating in a radio show and then wanting to pursue journalism or being inspired by a psychologist and pursuing psychology) [13]. Similarly, in an intervention on HIV prevention in Malawi, where youth were discouraged by their peers from participating in the HIV program, it was older adults and NGO personnel who encouraged them to continue. The second role was their support in building pressure for the “cause” of the group – for instance, providing legitimacy in meetings with the government, or supporting their demands in larger forums. Macwan highlights the importance of allying with adults in the community to lend more legitimacy to the youth groups. Oftentimes, given that SRH is a sensitive issue, parents need to be on-boarded. In some cases, this led to greater communication with children on SRH issues, which ultimately made them allies in negotiation with policy makers/health service providers [25, 29]. However, in situations where youth are included in groups with adults, it is also important to keep in mind cultural barriers and hierarchies that prevent, especially young women, from participating [14, 20]. 

To summarise, the articles mention the use of various context-dependent strategies to build pressure. How an issue is framed is of critical importance, especially given the sensitive nature of a subject such as youth SRH, and the means of influencing public opinion and discourse must suit the intended audience. Forging alliances both with those who are similar to the collective, but also casting a wide net of allies from varying backgrounds and interests, can strengthen collective action and help to include issues of other constituencies that can strengthen the network.

#### Negotiation with power holders/duty bearers

The third strategy is that of negotiation with powerholders, using a range of instruments depending on the demands and the context. The interventions varied both in the kinds of demands they were making, the nature of powerholders themselves (state/community leaders), and the policy and political context in which these efforts unfolded.

##### Leveraging existing policies

The presence of laws, policies and programs is an important feature in facilitating interventions, but being cognizant of their limitations is important. For instance, there is a recognition of youth participation as a right in the constitution and in policies in South Africa [16], which makes it imperative for youth to be involved in policy-making. However, the South African process described by Jacobs does not include youth in all aspects of the policy cycle. Moreover, past HIV donor-driven notions of youth participation in South Africa aimed at creating young women as girl ambassadors and role models, without substantive engagement with or accountability to the larger body of youth [16]. Similarly, in India, there is a National Adolescent Health Policy which lays out entitlements for SRH that can be demanded through collective action [25]. In Malawi, too, there is a place in policy and program at different levels for youth to participate, but this is restricted to policy consultations [19]. 

##### Utilising “invited spaces”

An important avenue where youth participation has been promoted has been in decision-making spaces with the purpose of making policies and programs more effective, for instance, in Malawi. In this case, youth are invited into policy spaces, but they often feel like their voices are not heard. In the case of Malawi [18] where a space for youth is guaranteed in all policy-making spaces, from village to district to national, youth solicited feedback about the program from peers, but felt that their participation was restricted. On the other hand, adult policy-makers and government officials think that youth contribute a lot. However, making allies within the health system can also help to bring about change from within, as was seen by Macwan et al., who found changes at the level of primary care after consistent engagement with individual health care providers [25]. 

At the same time, there is also the danger that facilities may deflect responsibility upstream, making multilevel engagement necessary. Similarly, in the case of a long-term intervention led by CARE in Malawi, which had transitioned to youth leadership, the most significant challenge that youth faced was in convening interface meetings with stakeholders, especially due to the lack of involvement from officials and duty bearers, unless this was facilitated by the NGO [26]. 

##### Navigating political opportunities

Political contexts also play an important role. In describing the evolution of the movement for sexual health policies in Ecuador and Peru, Coe describes three national level conditions shaping collective action: the development of progressive social movements and ideologies (both Peru and Ecuador saw progressive movements in the 1970 s after transition to democracy, but Fujimori’s counterinsurgency efforts repressed Peruvian democratic movements in the late 1980 s and 1990 s), favourable sexual and reproductive rights policies (recognized in both countries but less so in Peru due to the influence of the church and rise in neoliberal policies following Fujimori), and institutional support for adolescent health programs (autonomy to decide number of children was recognized in both countries but formal recognition of young people’s SRH rights existed only in Ecuador. By contrast, in Peru, sexual relations between boys and girls aged 14–18 were criminalised in 2006). Although both countries had cases of youth collective action around sexual health, only Ecuador was able to achieve a successful national advocacy campaign. Catholic conservatism in Peru, on the other hand, stymied efforts in that country [13]. There is also a role for making use of emerging opportunities on which to piggyback SRH rights agendas. For instance, in the case of Rwanda, the youth movement pushed through abortion reform when the revision of the penal code was underway, which provided a gateway into making amendments. Since the pressure had been built up systematically, the opportunity could be taken advantage of [11]. 

##### Influencing non-state power holders

In two cases [21, 29], collective action efforts were primarily directed at power holders within the community. Lofton et al. describe an intervention using photovoice methodologies for youth, which drove community changes to prevent HIV in Malawi through demanding changes from community leaders. For instance, youth noted that the opportunity to engage in “risky sexual behaviour” presented itself after church events and hence suggested that leaders avoid organising night prayers and could instead hold prayers during the day. Further, liquor shops and restaurants were seen as spaces where such encounters took place, and the youth demanded their early closure. While in the former, despite pushback from church leaders, change was possible, in the case of the latter, due to economic interest involved, no change took place. In another instance where an action research project sought to change community attitudes around rape in a Jewish community in New York [21], the youth group used a familiar community structure, in this case the Jewish feast of Passover Seder – reimagined for a new issue to trigger community reflection.

In summary, the evidence suggests that negotiation with powerholders in collective action interventions must be strategic and consider local contexts of policymaking. Not just the state, other powerholders in the community could also be held accountable. Moreover, even in contexts where policies that prescribe youth engagement are present, the readiness of the system to respond to and grant legitimacy to youth voices is necessary for effective implementation.

## Discussion

This scoping review summarises the literature available on youth, SRH rights, and collective action for accountability. It highlights that a wide variety of interventions/strategies can be considered within the ambit of collective action for accountability, their conceptual underpinnings and what enables or hinders the strategies that they use. The interventions broadly involve strategies to build collectives and platforms for youth engagement, build pressure through the use of evidence and allyship, and negotiate with power holders to bring about change. Within these strategies, there are various levels of depth and sophistication with which the interventions operate. Within building collectives, they vary from creating youth groups to leveraging the diversity of youth citizenry, to the extent of critical consciousness within the groups. Within their tactical repertoire to build pressure for their demands, they use tools such as scorecards or photovoice to a diversity of strategies that try to alter the narrative and discourse. Within negotiating with power holders, from utilising existing policies and invited spaces, to leveraging political opportunities and influencing non-state power holders. The scope of the change that they envision also ranges from narrow, i.e. addressing specific SRH issues, to broad-based social movements seeking to effect changes in sexual politics and democratic participation.

Based on this overview, we can distinguish a few challenges/opportunities for today’s expanding field of youth collective action for accountability towards SRH rights.

### The role of context

As is the case in complex interventions, the way strategies are implemented either by external institutions or by activists themselves, depends on the context within which the intervention operates. The context that influences the intervention consists of not just the micro, proximal factors at the grassroots level, but also includes meso- and macro factors, linked to socio-political realities (barriers and opportunities) as well. Social and cultural norms, both related to gender, as well as age, class and social hierarchies, have a deep and lasting impact on whether and how the SRH issues of youth are expressed and tackled. Structural drivers of health systems, social movements, political systems, and politics itself have a causal power over the “success” of interventions. The strategies must therefore be flexible, creative, and responsive to these contextual conditions. Historical analysis, which places the intervention within a longer history of civic engagement in that region, has not been done sufficiently in the literature. This has been the case more broadly in global health, which lacks a focus on historical complexity while analysing “successes” of global health endeavours [36, 37].

### Dangers of co-optation and backlash

With the growing interest in working with “youth”, it is important to cast a critical eye on these interventions and the nature of change that they are seeking to bring about. Youth engagement in SRH runs the risk of being used instrumentally in programs to increase utilisation of services, efficiency of interventions or risk being tokenistic [38]. While some of the interventions, focused on specific SRH issues, initiate groups with a specific goal in mind, like preventing HIV transmission or addressing adolescent pregnancies, the need for a broad-based youth citizenry has been highlighted as well [16]. Youth are not a homogeneous category, and it is erroneous to assume that they have common unified aspirations, as is sometimes projected on them. “Problem analysis” depends greatly on the lens that youth collectives use to view their world, and their own lived experiences. A broader social justice/social change approach that addresses longer-term structural injustice, rather than narrowly focused interventions to address specific SRH problems, is preferable. In fact, literature in youth studies warns about the governmentality of the category of “youth” [39], urging practitioners and researchers to look beyond limited aims of development programs that imagine “empowerment” in an instrumentalist manner that places the entire locus of control and choice on the individual, thereby distracting from larger structural issues that youth and others are affected by [35]. Engaging in such a process of critical analysis, reflection and action, however, requires sustained engagement with youth groups over several years, which externally funded interventions rarely allow. Further, there are challenges in ensuring that youth engagement is substantive. Frameworks such as Hart’s Ladder of Participation (drawing on Arnstein’s Ladder of Citizen Participation), Wong’s typology of youth participation and the CHOICE “flower of participation” focused on SRH have enabled a differentiated understanding of the scope and depth of engagement and empowerment that youth experience through the roles they play in the policy cycle. However, this is not easily operationalised due to differences in values and goals of different constituencies and the funding ecosystem, which includes funders and implementers.

### Attention to intersectionality

Very few of the articles directly tackled the issue of intersectionality in organising youth. Yet, those that did provide significant insights for youth participation in collective action. Factors related to gender and cultural identity (like religion or class), class, ability, and sexual orientation influence power dynamics at every stage of collective action. They dictate the strategies that can be employed as they relate to (1) barriers faced in collectivising of youth facing multiple social vulnerabilities (2), the collective’s identity (3), how issues are framed for public/social advocacy, and (4) the imperative to forge wide-ranging plural allies from different backgrounds. These make a compelling case for why interventions must adopt an intersectional lens.

### Limitations

The breadth of interventions that fall under the umbrella of “collective action for accountability” made it challenging to distil a unified theory of change. However, we have attempted to draw lessons for youth collective action based on the different strategies that these diverse interventions deployed. Given that real-world collective action is diverse and complex, we believe this is a valuable contribution to our field. A significant limitation of this review is that the analysis is restricted to the information provided by authors in their articles or reports. The lack of consistency and depth in reporting, especially in public health journals, does not allow for a deeper analysis of conceptual underpinnings, the content and process of the interventions. This is especially relevant to this review because of the non-linear and iterative nature of such interventions.

## Conclusions

Thirty years after ICPD, youth participation in sexual and reproductive health continues to be a critical area of action and research. In a changing world, youth collective action efforts are growing, both in the field of SRH and beyond. This review provides insights into how some of these efforts bring about change, as well as the risks and opportunities they pose. While policies to include youth in decision-making are necessary, there are a host of factors that determine the readiness of the system to realise them. Beyond engaging with the state (or state actors), the articles included in this review describe the myriad efforts that youth collectives make to effect change not just in policy and health services, but in society and discourse at large. Lessons from the review, especially the emphasis on adopting contextually relevant strategies that address and involve diverse youth, can guide future action in the field.

## Supplementary Information

Supplementary Material 1. Prisma Checklist for Scoping Reviews.

Supplementary Material 2. Electronic Search Strategy.

Supplementary Material 3. Table on Articles.

## Data Availability

The review uses published articles as the source of data. All references to articles have been provided.

## Footnotes

1 We refer to these as “interventions” which may imply that they have been induced by an external agency. However, these “interventions” represent both organically emerging efforts as well as those that have been deliberately implemented.
