# Supplementary material for: Youth collective action for accountability towards sexual and reproductive health (SRH) rights: a systematic scoping review
Source: BMC Public Health. 2026 Feb 26;26:1087. doi: 10.1186/s12889-026-26642-8 (PMC13041165; doi:10.1186/s12889-026-26642-8)
Supplement: Supplementary file 2 — Supplementary Material 2. Electronic Search Strategy. [file 12889_2026_26642_MOESM2_ESM.docx]

Wos 775: <https://www.webofscience.com/wos/woscc/summary/2cd51aa4-55e5-4760-bd64-7250d8bfde43-010ac0866b/relevance/1>

PubMed: (("2001/01/01"[Date - Publication] : "3000"[Date - Publication])) AND (English[Language]) AND ("adolescen*"[All Fields] OR Adolescent Health [MeSH] OR "youth" [TiAb] OR "young people" [TiAb]) AND ("community participation"[MeSH Terms] OR "youth participation"[All Fields] OR "community action"[All fields] OR "youth engagement" [All Fields] OR "youth empowerment"[All Fields] OR "citizenship" [All Fields] OR "accountability" [All Fields] OR "social accountability" [All Fields] OR "citizen-led" [All Fields] OR "collective action" [All Fields] OR "activism" [All Fields]) AND ("sexual and reproductive health" [All Fields] OR "sexual and reproductive rights"[All Fields] OR Reproductive Health [MeSH] OR Sexual Health [MeSH] OR Contraception [MeSH], Sexually Transmitted Diseases [MeSH] OR HIV OR STI OR "sexually transmitted infection" [All Fields] OR reproductive [TiAb] OR "sexual health" [TiAb] OR "family planning" [All Fields] OR contracept* OR abortion OR "early marriage" OR "violence against women" OR "sexual harassment" OR "Rape" OR "gender")

Web of Sceince: (ALL=(adolescent) OR ALL=(adolescent health) OR ALL=(youth) OR ALL=(young people)) AND (ALL=("collective action") OR ALL=("community participation") OR ALL=("youth participation") OR ALL=("community action") OR ALL=("youth engagement")OR ALL=("youth empowerment") OR ALL=("youth participation") OR ALL=("YPAR") OR ALL=(citizenship) OR ALL=(accountability) OR ALL=("social accountability") OR ALL=("citizen-led") OR ALL=(activism) OR ALL=(movements) OR ALL=("youth movements") OR ALL =("feminist movements") OR ALL=("social movement")) AND (ALL=(SRHR) OR ALL=("sexual and reproductive health")OR ALL=("sexual and reproductive rights") OR ALL=(Reproductive Health) OR ALL=(Sexual Health)OR ALL=(Contraception)OR ALL=(Sexually Transmitted Diseases)OR ALL=(sexually transmitted infection)OR ALL=(HIV)OR ALL=(STI)OR ALL=(reproductive)OR ALL=(sexual health)OR ALL=(family planning)OR ALL=(abortion)OR ALL=(early marriage)OR ALL=(rape))
