# Supplementary material for: Youth collective action for accountability towards sexual and reproductive health (SRH) rights: a systematic scoping review
Source: BMC Public Health. 2026 Feb 26;26:1087. doi: 10.1186/s12889-026-26642-8 (PMC13041165; doi:10.1186/s12889-026-26642-8)
Supplement: Supplementary file 3 — Supplementary Material 3. Table on Articles. [file 12889_2026_26642_MOESM3_ESM.docx]

| **Sr. no** | **Title** | **Year** | **Citation** | **Country** | **Intervention** | **Research methodology** | **Theoretical lens** | **Learnings for the review** |
| --- | --- | --- | --- | --- | --- | --- | --- | --- |
| 1. | Youth Action Research for Prevention: A Multi-level Intervention Designed to Increase Efficacy and Empowerment Among Urban Youth | 2009 | (10) | United States | Youth Action Research for Prevention (YARP)- (1) begins with individual training consisting of building relationships, exploring identity, assessing learning styles, and introduction to group and cooperative learning process (2) forges group identity and cohesion (3) trains youth as a group to use research to understand their community better and analyse problems from critically from a historical, structural and equity lens (4) engages them in using the research for social action at multiple levels in community settings (policy, school-based, parental etc.) The groups used findings from the research to create a PSA, a game and a play to engage families and peers in conversations around factors that lead to risky sexual behaviour. | Mixed methods, quantitative quasi-experimental design and qualitative by observing and documenting youth  activities and the responses of various target audiences and collaborators. | The authors justify that 14-17 years is a developmental period when adolescents seek more autonomy and prioritise peer opinions and relationships - this makes it a good point to intervene, especially with regard to identity building in case of marginalised groups. The intervention draws on critical theories, including the work of Foucault (to understand power), Bourdieu (to understand structures of oppression), Freire's (to articulate the right of people living under oppression to conduct a transformative analysis of their own reality, leading to change), and Gramsci (to highlight the importance of maintaining "hope"). | - Individual level training addresses exploration of identity and relationships, triggers self-critical self-analysis of attitudes and behaviours, leading to possibilities for changed attitudes and behaviours. It also encourages critical analysis of problems from an equity lens.  - The Group provides solidarity and positive interdependence leading to social cohesion. Cohesion leads to a sense of group efficacy to be able to work together to bring about change.  - Youth built evidence on issues that they chose and saw as priority issues. The research helped the groups to enable critical self-reflection among peers and families, which influences internal decision-making.  - Advocacy using research findings triggers a sense of an internal locus of control and improved social and assertive skills, leading to improved decision-making. The project led to some reduction in sex with multiple partners in the intervention group as compared to the control group, but this was not statistically significant. The authors emphasise that the intervention is designed to influence internalised decision-making, which requires time to take hold.  - The project was implemented with a community that is predominantly Latino or African/Caribbean- American, and many of them are poor or lower-income working class. The city is described as a "small city with big problems", referring to the structural and systematic issues of disinvestment, poor schools, lack of affordable housing and so on. The environment of the city decided the kinds of issues that the youth took up for PAR. - The authors emphasise the importance of a process like PAR for youth in a community facing historical problems in terms of both analysing their problems as well as the value of building supportive relationships and group action. |
| 2. | a. Youth participation in the fight against AIDS in South Africa: from policy to practice  b. From Rhetoric to Reality? Putting HIV and AIDS rights talk into  practice in a South African rural community | 2009, 2014 | (23, 24) | South Africa | This article examines the inclusion of youth participation in an existing project aimed at HIV prevention and AIDS care management (which included specifically involving youth in rallies, building capacity for peer support and counselling and facilitating participation in public life). | Qualitative methods, 105 in-depth, semi-structured interviews, with young people, church leaders, teachers, government officials, home-based caregivers and local leaders; and 52 focus-group discussions (with a total of 313 participants) including young men and women, home-based carers, local teachers in the community and traditional leaders  (indunas). Fieldworker diaries kept by project workers. | Recognising youth participation as an important condition for democratic renewal, the authors differentiate between a “narrow” understanding of participation (ie. to improve service delivery), versus a “broader” understanding, which is to empower youth and foster collective social action that addresses their exclusion. In this context, the paper examines the engagement of youth in a project on HIV prevention and AIDS management, using their “AIDS competence framework” to highlight the psycho-social preconditions for effective participation. | The project took efforts to ensure that its activities were engaging and relevant for youth and strived to provide safe spaces for young people to talk about HIV/AIDS. It recognised that young people were sexually active, despite opposition from the communities where it was embedded.  - The project’s success was obstructed by certain structural factors, such as entrenched poverty and unemployment, which made it difficult for youth to participate in its activities.  - Another structural factor was stigma towards HIV/AIDS, which made young people reluctant to take ownership of the issue.  - Social attitudes towards sexuality in general became a barrier to having open conversations with adults about HIV/AIDS, since the latter encouraged abstinence.  - The unwillingness of adults to recognise the potential and relevance of youth participation as equals was a barrier that the authors felt should have been tackled better by acknowledging the power dynamics between adults and youth, especially in a context where both are marginalised.  - The lack of support for other external agencies towards youth participation also served as an obstacle.  - Finally, the lack of meaningful incentives for youth to participate could have been addressed better.  - The papers summarise that “youth are most likely to participate in social development projects when they have the appropriate knowledge, social spaces for critical thinking, a sense of ownership of the problem in question, a sense of confidence in their ability to contribute to solving it, and appropriate bridging relationships.” |
| 3. | Maternal health challenges experienced by adolescents; could community score cards address them? A case study of Kibuku District– Uganda | 2020 | (20) | Uganda | Community Score Card approach, which brings together service users and providers to address problems and find feasible solutions. A CSC tool to assess facilities is used as a basis for discussion, which is prepared in a participatory manner. Adolescents were included as a target group in these meetings. | Qualitative methods primarily using IDIs with 15 purposively selected adolescents who had attended CSC meetings and given birth 2 years prior to the study. | No specific conceptual theory, but the study identifies Adolescents as a vulnerable group vis-à-vis maternal health and the inability of health workers to address the specific problems of adolescent pregnant girls. It hypothesizes that social accountability tools such as Community Score Card (CSC) would help to bring out the voices of adolescents and help to address them. | - Adolescents were a target group and were expected to participate in the community meetings, but were unable to do so, due to being in school. Even those who did attend were quiet because they did not want to speak about issues related to sexuality in public.  - CSC tool was developed, and priority areas were scored by the group. But the lack of participation of adolescents meant that their issues did not get incorporated into the community scorecard's priority issues.  - Broader issues affecting maternal health could be addressed, but not those specific to adolescent issues, such as rejection by parents and partners, lack of birth preparedness, legal support, etc. |
| 4. | Enhancing social accountability through adolescent and youth leadership: a case study on sexual and reproductive health from Gujarat, India | 2021 | (25) | India | -Capacity building of peer leaders to understand their entitlements  - Creating collectives at the local level under the leadership of the peer leaders.  -Building support structures through parents’ groups and local advisory committees at the village level  - Sensitising and strengthening relations with health service providers  - Monitoring by peer leaders and demands for change in the quality of services | Qualitative study including review of program reports, interviews | - Gender relations are power relations and must be transformed.  - Youth are potential leaders and responsible citizens. Through social accountability, they can make collective demands for entitlements.  - Youth have needs that are different from adults, and they have the right to personal development, information and opportunities. They constitute a demographic dividend that can only be leveraged if healthy.  - Intersectionality perspective - recognising multiple layers of disadvantage among youth themselves. | - The process of the intervention has resulted in changes in social norms, like girls and boys being able to attend training together.  - Use of simple tools enabled monitoring of the quality of services.  - Local advisory group + parents+ service providers provide support to girls for advocacy. This has resulted in girls delaying their marriages, increasing their mobility outside their villages, continuing higher education, aspiring for good careers, and showing effective leadership.  - Existing policies and entitlements for adolescent and young people’s SRHR are in place and are leveraged as a part of citizenship rights.  - Peer leaders are being supported and responded to by different authorities: three a*nganwadi*s were repaired, and two received approval for funds, two PHCs began displaying services available for adolescents.  - Recognition that disabled youth, trans, and tribal youth have different issues. Diversity in NGO partners in the intervention – working with specific disabled, trans groups.  - Intervention adapted to address diverse needs, for example, adjusted criteria for trans peer leaders to 30 years because recognition of identity occurs late, and persons with locomotor disabilities are unable to travel, so they need to work with them individually or with parents at first. |
| 5. | a. Evaluating Young People’s Ability to Sustain an Evidence-Based Social Accountability Approach to Improve Adolescent Sexual and Reproductive Health in Ntcheu, Malawi  b. From effectiveness to sustainability: understanding the impact of CARE’s Community Score Card© social accountability approach in Ntcheu, Malawi | 2021 | (26,27) | Malawi | CSC intervention in Malawi through the Maternal Health Action Project – CARE in partnership with Govt of Malawi to train “Comm health action groups” CHAGs to use scorecards to assess services by the community. These formed the basis of meetings with district-level officials and negotiations for improvement in services. Role of young people – they were involved in trainings, identified the need for a safe space to talk about their health issues. The organisation and the government established 5 youth clubs in the health centre catchment areas, which were envisioned as “a safe space for youth”. Young people in these clubs, who had previously been part of the health action groups, introduced the scorecard process to their peers in the clubs. | Qualitative descriptive study, evaluation used a PAR approach to understand how youth can sustain the intervention and challenges faced therein. | The article uses the lens of sustainability to explain the importance of engaging young people as: “initiatives driven by young people that change power dynamics, governance structures, and expectations of the relationship between government and  community have the potential to create fundamental, lasting structural change that improves health and development. Since young people are a dynamic, ever-changing segment of the population, it is important to understand their ability to pass along knowledge and ensure sustainability.” | - The authors saw a diffusion of the original CSC intervention among youth, evidenced by the fact that young people organically adopted aspects of SRHR that were important to them, such as child marriage and girls' education. They also emphasise that it was the youth who trained each other in the CSC process and recommended a nationwide scale-up.  - The project helped young people in Ntcheu take a more active role in the community and in development projects that affect them. It helped them gain respect within their communities.  Stakeholders agree that the provision of a safe space for youth is important.  - Through the project, youth were empowered to speak freely, with transparency, and with accountability.  - Convening and facilitating interface meetings with stakeholders was a challenge for young people due to a lack of involvement from officials and duty bearers, and the absence of CARE to serve as a broker and facilitate these meetings.  - Problem of resources – for refreshments, transportation, flipcharts, notebooks. For future such endeavours, the authors suggest a transition period in which periodic refresher trainings are held and a small fund to support implementation is made available. |
| 6. | a. Developing Action Plans in Youth Photovoice to Address Community-Level HIV Risk in Rural Malawi  b. “Youth Photovoice”: Promoting Youth-Driven Community Changes for HIV Prevention in Rural Malawi | 2020 &  2021 | (28, 29) | Malawi | The project takes a community-based participatory approach using photovoice, a community-engaged, visually based methodology. It was implemented as part of a peer group program for HIV prevention in three communities. Eight sessions were held with 24 youth aged 13-17 (12 males and 12 females). The youth took photographs of places or situations that “encourage or discourage risky sexual behaviour”: initiation ceremonies, isolated areas, community celebrations, local businesses such as bars and rest houses, and church-sponsored activities. The youth then developed action plans through facilitated discussions and presented them to the community. | Qualitative methods, 4 focus groups: two with the Youth Photovoice participants, one with parents, and one with local community leaders. 5 interviews with the adult volunteers and 2 community leaders, field observations and recorded transcripts of photovoice sessions. Data sources also included photographs selected by the youth, the co-created analysis of those photographs, and the detailed action plans that they presented. | HIV prevention often addresses individual behaviour change, without considering community-level factors. This understanding informs the project's focus on addressing such factors that promote “high-risk environments”.  The authors suggest that photovoice as a method is “developmentally appropriate” and engaging. It allows youth to “take ownership over the research by (1) empowering youth to identify and reflect on community assets and challenges, (2) encouraging critical dialogue, and (3) creating opportunities to develop a plan to inform and reach those with power.” | - The goal was mainly HIV prevention through addressing “risky” sexual behaviours (a narrow goal). What constitutes risky behaviours was not interrogated.  - “Risky environments” were identified by youth as initiation ceremonies, isolated spaces, late evening church events, presence in bars and pubs, who made a plan of action, presented to and negotiated with leaders (with sympathetic adults as allies) - adults see the rationale and are convinced, leading to a change in practice.  - The group process followed a “single-gender” approach because, in their experience, the implementers saw that single-gender groups allow for greater participation by young women.  - The group process facilitated cohesion, engagement, and inclusiveness and created a “safe space” to discuss sensitive issues.  - The project challenged prevailing social norms around youth participation and adult-youth dynamics by making sure that the process was youth-led, particularly in an HIV prevention initiative.  - The youth faced discouragement from their peers (who suggested they were wasting their time for no benefit) and from some parents (who referred to them as “orphans,” implying that their parents succumbed to AIDS, suggesting stigma). The youth discussed their discouragement with their own parents and staff, who provided encouragement and helped them to navigate the negative responses.  - Resistance from a few community members who did not want to change their traditional practices, such as moving initiation ceremonies from the bush to church settings. But community leaders were broadly supportive and encouraging.  - Factors influencing advocacy - broader socioeconomic and political context to the challenges that the youth face. Non-economic issues, such as changing the timing of events, were generally supported by the community and were addressed. However, those where economic interests were involved (such as changing practices of alcohol stores and rest houses) were not successful because they threatened revenue and potentially pitted them against larger marketing campaigns, such as those driven by the alcoholic beverage industry. |
| 7. | Feminist Activism and YPAR: Privileged Girls Interrupt Rape Culture | 2020 | (21) | United States | Uses YPAR as a tool.  Bi-monthly sessions in feminist theoretical and social justice education, and the design and implementation of participatory action research, were held with high school students, through which youth came to understand that rape culture has deep roots and “disrupting it depends on naming its reality within their lives and its systemic foundations.”  Building on this they took steps to educate their community about rape culture and gender-based violence and created strategies to transform rape culture and facilitate social change within their own community. | Qualitative documentation of the intervention and interviews. Two domains of inquiry:  (a) uses of YPAR, in particular, with privileged youth; and (b) youth feminist-informed activism challenging rape culture | Critical, intersectional feminist  principles and community-engaged action toward social justice – direct attention to the root causes of social problems. | - Collaborative critical inquiry - YPAR pedagogy creates an environment in which young people can intentionally draw on a feminist approach to interrogation and critique through which to locate themselves within intersecting  axes of privilege and oppression.  - Engaged youth from backgrounds of relative race and class privilege to critically interrogate how they may be simultaneously impacted by systemic privilege and oppression, and in the process, reevaluate their beliefs about themselves, others, and the world.  - Naming reality within their own lives and exploring foundations of the problem- youth were able to discuss and frame a social problem that they knew, intuitively, existed, but didn’t necessarily have a language and critical analytic tools to interrogate - what had been hidden and invisible now had the opportunity to surface and become illuminated.  - Teaching students of privilege to become critically conscious and take steps to challenge the status quo, to be an accomplice rather than an ally – someone willing to take a risk to upset the status quo.  - Use of community rituals –participants took up the topic of rape culture within the Jewish community at the Passover Haggadah and community Seder, a ritual with which their community is very familiar. This facilitated an internal reflection in a context that the community is familiar with. |
| 8. | Evaluation of a youth-led participatory action  research to address adolescent sexual and  reproductive health and rights issues in Senegal | 2022 | (30) | Senegal | Participatory Action Research – groups of young men and women selected with community consultation, assigned an adult mentor, received systematic inputs on research methods and SRHR. They conducted small research projects and disseminated findings to the community.  Includes:  Training and practice in research techniques  Practice strategic thinking and discuss strategies to influence change  Building support networks in collaboration with stakeholders  Power sharing between adults and young people in the process of research and action, as well as among youth participants themselves.  Opportunities and advice for working in groups in order to achieve the expected  objectives  Development of skills to communicate with other adult and young actors | Qualitative interviews with participants, mentors and community members | Recognition that the development of effective adolescent programming requires the involvement of adolescents themselves. The involvement of adolescent girls is identified as an ethical and human rights imperative.  Evaluation draws on YPAR principles articulated by Villa-Torres and Svanemyr (2015) and on the Conceptual Framework for Measuring Outcomes of Adolescent Participation from UNICEF (2018). | - Adolescent outcomes include increased sense of self-worth, self-esteem and self-efficacy, making decisions, being taken seriously and public and civic engagement.  - Trainings included an overview of ASRHR issues in Senegal, but the participants felt this was too limited, as they did not have sufficient background information on a technical topic. (balance between technical versus lived experience, and which to prioritise in PAR)  - Groups were mixed to create trust between diverse groups – ethnicities, genders, ages, married/unmarried.  - Adult mentors stayed in the project area for the duration of the project, and this helped to develop meaningful relationships between the mentors and participants.  - In a conservative context where there is little conversation about SRHR, the context of a research project allowed more conversation in public, but still not between adolescents and their parents.  - Adolescents reported an increased sense of self-worth, self-esteem and self-efficacy vis-à-vis identifying solutions and communicating their point of view. Some who had left school expressed a desire to resume studies  - Perception of community members that if the research had been done by adults, it would have had more weight, as community health workers, for example, are unwilling to listen to young people.  - According to adults, adolescents were autonomous, but according to adolescents, they had autonomy in choosing a research topic and conducting the research themselves but followed the instructions of the mentor. |
| 9. | Evaluating the feasibility of the Community Score Card and subsequent contraceptive behaviour in Kisumu, Kenya | 2022 | (9) | Kenya | The youth groups monitor and evaluate the quality of services using the Community Score Card (CSC). The groups (1) facilitate identification of priority areas of improvement through meetings with the community, (2) create corresponding indicators to measure them, and (3) monitor the quality of services using the indicators. (4) After monitoring, the community and service providers hold a dialogue to discuss the findings and create a concrete plan along with a timeline for improvement. (5) This plan is then monitored by the groups once again. | Mixed methods - Quantitative methods including mystery client visits, unannounced visits, repeated assessment of score card indicators, and service delivery statistics. Qualitative data - focus group discussions with youth working group members and providers at intervention facilities. | Access to contraception among young, unmarried women is hampered by provider attitudes. A Youth Working Group (YWG) would give voice to the problems faced by youth in accessing facilities. | - CSC implementation approach was developed and led by Youth Working Groups consisting of 4-6 youth between the ages of 18 and 30 years. Groups held meetings with other youth, which were supposed to be about CSC implementation, but ended up also including education and conversations around family planning since there was a demand for information among youth. It appears that this was a requirement before implementing the CSC.  - Parents of youth were reluctant to send their children to the youth groups, because in the past, some children had been given contraceptives without the parents' consent. Hence, the youth groups used community health volunteer support to gain the parents' trust.  - Priority areas identified by community and service providers in the development of the Community score card were used to build evidence around the status of facilities and experiences of users, which did not completely overlap.  - A space for dialogue was created in the facility, where the community and providers discussed issues and made a plan.  Some issues were improved upon, such as the behaviour of providers and informal payments, but issues related to stockouts or providers being overworked could not be addressed. Some providers saw the CSC as a way to educate patients rather than as a tool that was meant to monitor them. |
| 10. | Youth-Led Social Accountability Interventions: Get Up Speak Out (GUSO) Alliance | 2018-2022 | (31-33) | Ghana, Ethiopia, Malawi | Youth-led social accountability project implemented by a consortium of international organisations and local partner organisations. Different types of social accountability interventions are used. These include community scorecards and client feedback forms. They involve (1) training youth on SRHR, (2) developing indicators/tools, (3) implementing scorecards and other tools, (4) interfacing with health care providers and officials, (5) developing action plans and follow-up.  Some variations – Malawi used a mystery client approach while the other two sites did not. | Qualitative methods - focus group discussions and interviews with young women and men who were involved in the intervention and those who were not, service providers, staff of the implementing organisations, other people involved in social accountability activities, representing communities, schools, and district-level government departments. | The intervention aims to reach and effectively engage young people  to understand and demand their sexual, reproductive and health rights (SRHR). It uses a social accountability approach, ie, community-led efforts to demand accountability for guaranteed rights and entitlements. | - The policy context is variable in the three countries – in Malawi, there are criteria for health services to be youth-friendly, specified by the Ministry of Health. These were used as a standard for assessment.  - Youth reported feeling confident, able to articulate their concerns, and understand SRHR issues.  -Youth felt like the issues in the scorecard represented their concerns since they had been involved in developing them.  - There were barriers, such as some providers being judgmental, but most were responsive. Parents/elders not supportive – stigma about being seen around youth facilities  - Health providers reported that the assessment of services helped them understand the needs and problems of youth better and address them.  - Reported changes in facilities include greater youth friendliness and greater availability of contraceptives for youth. In some cases, although action plans were made, there was no framework for follow-up.  - The diversity of youth represented was variable. While the project in Ethiopia included people living with a disability, in- and out-of-school youth and female sex workers, the one in Ghana leaves out youth who cannot read and write, who are many. |
| 11. | a. Understanding How Young People Do Activism: Youth Strategies on Sexual Health in Ecuador and Peru  b. How gender hierarchies matter in youth activism: young people's mobilising around sexual health in Ecuador and Peru | 2015 & 2013 | (12, 13) | Ecuador/Peru | Advocacy networks/Social movements- 4 cases of collective action (60 youth were interviewed, most aged between 15 and 25)  1. Developing individual activists  2. Developing young people's collectives/organisations/networks  3. Building alliances with other youth organisations and other adult organisations  4. Social Advocacy - campaigns, radio programs, bringing "emotion" into serious messaging, using culture - imp - challenging accepted gatekeepers of messaging such as schools, businesses, other adult organisations  5. Policy Advocacy - being part of committees of the government, joining political parties, pushing for new policies for SRHR, participating in the drafting of the new constitution  Commonality along five interconnected strategies, none of which was developed entirely or intentionally from the outset but rather emerged in the development of young activists’ collective action. | Qualitative study, using a case study and grounded theory approach, which allows youth to articulate their own definitions of their collective action. | Builds on the concept of “tactical repertoire” (Tilly, 2008), which refers to the set of protest tactics used by new movements making claims upon the state. However, movements do not continuously use protest tactics, nor do they constantly make claims of the state. Moreover, most youth do activism outside the formal political sphere. The study, therefore, seeks to build on concepts like tactical repertoires.  The second article draws on Taylor’s (1999) framework on how gender hierarchies intersect in social movements across opportunity structures, mobilising structures, and collective framing and identity. It examines how these apply to youth activism on sexual health. | - Youth define their collective action across four dimensions – the country dimension, which includes social movements and policies; the case dimension, which includes organisational scope and diversity; the strategy dimension, which includes the interconnectedness of the five strategies used; and the process dimension, which includes the construction of a youth identity.  - The article identifies three national conditions shaping youth’s collective action: (1) the development of progressive social movements and ideologies (both Peru and Ecuador saw progressive movements in the 70s after transition to democracy, but in Peru Fujimori's counterinsurgency efforts repressed democratic movements in the late 80s and 90. in Ecuador indigenous and class based democratic movement tended to flourish more), (2) favourable sexual and reproductive rights policies (recognized in both countries but less so in Peru due to a church lobby - influence of church and rise in neoliberal policies following Fujimori), and (3) institutional support for adolescent health programs ( autonomy to decide number of children recognized in both countries but explicit recognition of young people's SRHR only in Ecuador in 2006 - by contrast, in Peru sexual relations between boys and girls aged 14-18 criminalized in 2006).  - Alliance building/identity creation: developed relationships with youth from a wide range of geographical and social backgrounds and engaged with non-related adults in diverse ways.  - Adult allies – mentoring role - gave young people inspiration to pursue certain professions/vocations. (e.g., participating in a radio show and then wanting to pursue journalism, or being inspired by a psychologist and pursuing psychology)  - Policy advocacy: the five organisations in Pucallpa carried out an advocacy campaign to get a decree guaranteeing adolescents access to information to prevent pregnancy and STIs/HIV without parental consent. The regional government of Ucayali passed the decree but did not put it into effect due to interference from the Catholic Church.  - Gender and class hierarchies intersect with youth activism in that they determine access to and benefits of activism as well as supportive home environments (especially the control of adults over girls and non-control over boys). Within the organizations the activists could question dominant  gender norms and practices, while testing new ones, and challenge gender segregation and heteronormativity. |
| 12. | Advocating for safe abortion in Rwanda: how young people and the  personal stories of young women in prison brought about change | 2013 | (11) | Rwanda | This article describes how, through a project launched by Rutgers WPF on “sensitive issues in young people’s sexuality”, a process was initiated through which the abortion law was passed in Rwanda in 2012. It describes how the Rwandan Youth Action Movement decided to work on unsafe abortion as part of this project, after a study visit organised by Rutgers to the Netherlands. They 1) gathered data on the extent of unsafe abortion and testimonies of young Rwandan women in prison for abortions;  2) organised debates, values clarification exercises, interviews and a survey in four universities; launched a petition for law reform;  3) produced awareness-raising materials;  4) worked with the media and representatives from government ministries, the national women’s and youth councils, and parliamentarians. This played a significant role in the advocacy process for the amendment of the law, which was revised when the penal code came up for review in June 2012. | Qualitative – based on the authors’ own experience of advocacy for the law | Young people have the right to comprehensive information and services that promote their sexual and reproductive wellbeing,  including safe abortion services. This right is guaranteed by international human rights treaties and emphasised in intergovernmental agreements such as ICPD and MDGs. | - A collective of youth from Rwanda who were exposed to reproductive rights discourse through a study tour and workshops with a Dutch organisation, initiated a campaign to decriminalize abortion in their own country.  -The main strategy was to generate empathy for women who had been incarcerated for seeking an abortion. For this, testimonies were collected, which gave a "face to the problem" and generated public debate on the problem. People understood that there was more to the “Crime” of abortion than the morality of the girl involved.  - Values clarification workshops with youth and allies- methodologies used were designed to provoke empathy and reflection in combination with factual knowledge provided by the testimonies.  - Partnering with Civil society organizations serving youth, with the greatest potential to influence public opinion and decision-making regarding abortion in Rwanda.  - Some people strongly opposed the legal reform. An important condition for success in achieving understanding during the workshops was that everybody was entitled to their own opinion, which should be respected.  - Policy makers who were sensitive to the cause, impressed by the involvement of youth.  - Efforts coincided with important policy events, like the revision of Rwanda’s penal code and the ratification of some international and regional conventions that supported the granting of women’s reproductive Rights, notably the Protocol to the African Charter on Human Rights and on the Rights of Women in Africa. |
| 13. | Being young and LGBT, what could be worse?’ Analysis of youth LGBT activism in Indonesia: challenges and ways forward | 2018 | (22) | Indonesia | The article describes the broad strategies of young LGBT organising in Indonesia. It includes the following:   - Responding to structural violence and rights abuses - Integrating youth LGBT issues into ‘mainstream’ organisations - Inclusion of sexuality education in different settings - Provision of health services for LGBT youth - Advocating a national identity card for trans people | Qualitative methods - 46 people were interviewed either face-to-face or  through Skype/telephone conversations. | The interventions described are activist strategies of the LGBT movement.  The research is guided by a feminist intersectional perspective, which understands young people’s experience of being young and LGBT, along with other aspects of identity, such as location, which intersect with these. | - The article describes the hostile environment towards LGBT persons in Indonesia, including the growing backlash from neo-fundamentalist and conservative forces. This backlash takes the form of direct violence, strategic moves such as advocacy to pass legislation, as well as homophobic statements from public leaders.  - In this context of backlash, response to violence and provision of services to LGBT communities take precedence over other strategies because of their urgency and direct need.  - However, the authors highlight the importance of more strategic movement strategies, including mainstreaming the LGBT agenda, pushing for comprehensive sexuality education, integration of LGBT needs into youth clinics, addressing provider bias, campaigns by private sector entities to transform perceptions and so on.  - Apart from health professionals and teachers, the respondents find that an important overlooked stakeholder is parents.  - While “rights-based” demands might be considered “western”, an “anti-violence” approach may garner more support, as per activists.  - Challenges within the movement, especially for youth, include the bias against young people, which relegates them to lesser roles, as well as within-community hierarchies between urban-rural which has a bearing on funds and opportunities. |
| 14. | CrowdOutAIDS: crowdsourcing youth perspectives for action | 2013 | (15) | Global | A virtual participatory policy process implemented via an online platform, to develop a strategy for how to better engage young people in decision-making processes on  AIDS. 3,497 young people aged 15–29 from 79 countries signed up to nine online forums. In addition, volunteers recruited through the online platform hosted 39 community-based offline forums with an additional  1,605 participants.  The virtual space was intended to connect a community of interested young people, create a space where young people could share their experiences, ideas and information, synthesize knowledge and enable them to find solutions, and lastly, collective action through co-authoring the final strategy document, and the establishment of a network of youth activists and  organizations that the UNAIDS Secretariat could work with to implement the strategy. | Qualitative - data for this article were drawn from a secondary analysis of the themes and texts that emerged during both online and offline discussions | Human Rights and UN frameworks of the importance of youth participation as a right. | - There were similarities in the dataset across regions, both offline and online, in increasing communication about sex and sex education. However, differences were observed within forums from the same country, as well as in comparisons of offline and online groups. The major difference between the offline and online forums, in response to the “change one thing” question, was that offline forums emphasised cultural aspects of their community and the need for morals and more “responsible” behaviour, whereas online forums placed much greater emphasis on the need to open up communication and break the taboos surrounding issues of sex and relationships. Possibly – online participants had a higher socioeconomic background, were more globally exposed, had greater access to education, and perhaps did not feel the same weight of cultural pressures or more conservative ideas about moral standards and behaviours.  - Folly of thinking that all youth are “progressive”, but in fact they have mixed views. But an online forum allows more people to participate.  - Virtual tool opened the base of participation beyond youth networks and organisations that are traditionally consulted by global institutions, to involve “ordinary” young people who may never previously have been involved in sexual health and HIV activism. Individuals did not have to be formally organised or  part of a network or institution.  - Need to have both online and offline forums – to ensure that those who do not have digital tools can participate |
| 15. | The wave of the future? Youth advocacy at the  nexus of population and climate change | 2014 | (17) | Global | The article tracks the practices through which young climate change activists engage demographic-climate studies and broader development discourses as a basis for advocacy to influence international population and family planning policies. To illustrate this, it uses the example of a group of transnational youth during a workshop at the sixth annual Climate Change Conference of Youth (COY) as well as training workshops in the USA, exploring logics and discursive strategies employed by them. | Ethnographic observations of young transnational advocates during workshops and conferences. | The author uses a critical lens of technicalisation of women’s rights – “a set of strategic translations,  or reductions, shifting such complex issues as gender inequality and lack of reproductive health services into the narrow category of technology access” that is, contraceptives. This lens is used to interrogate the strategies underlying the promotion of youth advocates as important actors in “turning the tide on both global population growth and climate change”. | - Young population-climate advocates use the language of youth leadership to establish their activism as “innovative, youth centred and distant from the freighted, ugly politics associated with histories of population control.”  - Pragmatic approach - leveraging attention to climate change as an opening for increased attention to SRHR– people need access to contraceptives, and climate change is an easy sell, especially for youth  - Tactics are a simplified narrative, imperfect data made to fit a perfect problem statement, a quick solution, credible ambassadors, “positive” presentation (women’s empowerment, social justice, etc.).  - Youth advocacy reflects strategies and priorities of larger development institutions with which they were associated, and  ultimately led by.  - Geographic location of advocates and those they advocate for remains undefined, relegated to vague notions of ‘global’ contraceptive users. |
| 16. | a. Youth participation in sexual and reproductive health: policy, practice, and progress in Malawi  b. Participation of young women in sexual and reproductive health decision-making in Malawi: Local realities versus global rhetoric | 2020 & 2022 | (18, 19) | Malawi | These articles analyse and interpret young people’s lived experiences of participation (ranging from informal opportunities to formal structures) in SRH policymaking, especially how structural and societal factors influence their agency and participation. Participatory structures exist from community to village to district to regional to national to international. They aim to provide young people with space to solicit their peers’ priorities, share youth SRH experiences with local and national decision-makers, and provide feedback to youth in communities. Representatives for some structures are elected by peers (e.g., youth clubs vote at village/area/district levels). | Critical, focused ethnographic study based on document analysis, focus group discussions, and ‘‘moderate’’ participant observation, semi-structured interviews with key informants and youth (unmarried, without children), supplemented by open-ended drawing exercises with youth. Youth self-identified as either currently participating or not participating in SRH policymaking. The study included out-of-school youth or those experiencing homelessness. | The study is informed by post-colonial feminism and difference-centred citizenship theory.  Postcolonial feminism “exposes and deconstructs the influences of colonisation, racialisation, globalisation, gender, and  social relations that affect people’s lived experiences.” It challenges the subjugation and silencing of “Third World” youth experiences, which represent intersecting social categories.  Difference-centred citizenship theory “acknowledges that young people should be 'differently equal’ and not considered inferior to adults.” In doing so, it extends power to youth by recognising their differences (e.g., age, gender, class) as assets. | - Space in policy and program at different levels for youth to participate – but they do not consider it operationalised – mainly participation in policy consultations. On the other hand, adult policy makers and govt officials think youth contribute a lot.  - There was a compounding effect of social identities that determined the level and depth of participation. “Younger youth, females, people with limited formal education, and those living in rural areas have fewer opportunities and resources to engage meaningfully.”  - While most youth were engaged in grassroots or community-level entities, only elite, highly educated and experienced youth were selected to engage in policymaking forums at national and international levels.  - Gender bias – labelled *“prostitutes”* or *“whores”* by both adults and peers if they participate in SRH programs. Boys express jealousy that girls receive a lot of attention in development. |
| 17. | Aspirations versus Reality: Exploring Factors Affecting Meaningful Youth Participation in Sexual and Reproductive Health and Rights Programme in Rural Kenya | 2021 | (14) | Kenya | Inclusion of youth representatives in an existing participatory governance forum.  The project integrated youth representation in the Community Health Committees (CHCs) in 170 CHUs. Young people’s roles included managing computer-based SRHR information centres; conducting outreach services in collaboration with health facilities; advocacy with policymakers and implementers; undertaking household visits; conducting youth-led dialogue days where young people come together to discuss factors affecting their access to health care services; and running community campaigns to sensitise community members to support youth access to SRHR information and services. Youth groups met monthly to arrive at key issues to be presented to CHCs. | Qualitative methods - 29 key informant interviews and 13 focus group discussions conducted in five community health units across four counties. | Meaningful Youth Participation framework – ladder of participation in governance processes. | - Mismatch between expected and actual roles assigned to youth – typically they ended up engaged in health promotion or supportive roles assigned by the CHC leaders who were adult community representatives, local leaders or state officials – not accorded voting rights or key decision-making roles.  - Economic factors had a bearing on youth participation. Because participation was voluntary, those who lacked financial resources were often unable to meet logistical requirements such as travel. Moreover, due to unemployment, migration for jobs limited youth participation.  - Deeply ingrained principles of respect for elders manifest themselves in expectations of strict obedience and discipline, and young people occupying an inferior position to adults  - Change through invited spaces can be hindered by adult attitudes towards SRHR – especially attitudes towards girls or "undisciplined" youth, which leads to youth feeling powerless on boards.  - Those who were from clans other than that of the CHC leader faced greater barriers to participation. |
| 18. | Between Rhetoric and Reality: Learnings From Youth Participation in the Adolescent and Youth Health Policy in South Africa | 2022 | (16) | South Africa | The article analyses the youth participation process in policy development, which includes the following:  - Convening a Youth Health Parliament  - Visual exercises including ‘dream consultations’ and ‘dream clinics’  - Participatory research to investigate substance abuse, mental health/illness and adherence to chronic medicines  - Health clinic report cards in which adolescents and youth evaluated public health services  -Focus groups on sexual and reproductive health, intimacy, romance, risk and aspiration among youth and adolescents and their caregivers | Qualitative case study, interviews with policy actors. | The conceptual framework builds on an existing model drawn from fields of feminist, post-structural and critical theory, as well as youth studies, and citizenship research for conceptualising and planning for youth participation in programmes. The model directs attention towards seven interconnected domains of Purpose, Place, Process, Positioning, Protection, Perspective and Power relations. The authors add two additional domains of People and Partnerships to this model. | - Contextual factors – enabler - Participatory governance is an important right in the relatively recent democratisation in South Africa.  - History – post-Mbeki interest in involving adolescent girls and young women in HIV programming owing to data showing high burden in these groups – international initiatives – She Decides, DREAMS, She Conquers campaigns – youth participation mainly in the form of “ambassadors” which has set the tone for what youth participation means in South Africa. Some young people are treated as celebrity ambassadors, but without representation or accountability to the broader population of young people, nor attending to structural and systemic issues of youth disempowerment.  - Youth participation was enabled by leadership from key actors (in govt and academia) with a foundation in long-standing youth research participatory programmes.  - Challenge of diversity - when, how and which youth were involved.  - Inconsistent participation throughout the health policy formulation process.  - Siloed health priorities and policy processes, donor priorities  - Broader contextual challenges, including the lack of a representative and active youth citizenry. The youth sector is fragmented, includes some party-political structures, but no organised, nationally representative civil youth structures and movements.  - “An essential element for effective participation is a mobilised, capacitated, diverse youth citizenry as important actors to ensure youth participation, and the use of available tools and resources and guidance in a reflexive manner.” |
